# A Comparative Study of Two Types of Implantation Surgery Methods for Implantable Collamer Lenses

**DOI:** 10.1155/2021/4074773

**Published:** 2021-11-20

**Authors:** Hao Liu, Denghao Dong, Chunlin Chen, Jian Ye

**Affiliations:** Department of Ophthalmology, Daping Hospital, Army Medical University, Chongqing 400042, China

## Abstract

**Purpose:**

To investigate the effects of two different surgical methods of implantable collamer lens (ICL) implantation on the operation time, visual outcomes, corneal endothelial cell count, and intraocular pressure (IOP).

**Methods:**

This was a contralateral eye comparison study, a total of 192 eyes from 96 patients were included, and the two eyes from the same patient were randomly assigned to two groups (group 1 and group 2, with 96 eyes in each group). In group 1, after making the corneal incision, ophthalmic viscosurgical devices (OVDs) were first injected into the anterior chamber followed by ICL implantation. In group 2, the ICL was first implanted into the anterior chamber followed by OVDs injection. The operation time, uncorrected distance visual acuity, corrected distance visual acuity, spherical equivalent, corneal endothelial cell count, and IOP were recorded and analyzed.

**Results:**

The operative time in group 1 was significantly longer than that in group 2 (*P* = 0.002 < 0.05). There were significant differences between IOP measured 2 hours following surgery of the two groups (*P* = 0.026 < 0.05), Furthermore, the rate of IOP change 2 hours following the operation was significantly higher in group 1 than in group 2 (*P* = 0.019 < 0.05). There were significant differences in the anterior chamber angle 2 hours after surgery compared with that before surgery in both groups (*P* = 0.014 < 0.05 and *P* = 0.029 < 0.05, respectively). No significant differences were observed in the other parameters measured (all *P* > 0.05).

**Conclusion:**

The two ICL implantation methods had similar clinical outcomes and effects on the corneal endothelial cell count. Additionally, the implantation of an intraocular lens prior to injecting OVDs reduces the operation time and lowers the rate of IOP rise in the early postoperative period, making it safe and effective for ICL implantation.

## 1. Introduction

Correction methods for refractive errors mainly include corneal refractive surgery and intraocular refractive surgery, with the latter primarily being phakic intraocular lens (PIOL) implantation. PIOL implantation involves the placement of an intraocular lens into the eye to enhance the natural lens function without any manipulation. PIOLs are considered safe and effective for correcting high myopia [1] and provides many advantages including a speedy visual recovery, retention of the natural lens' regulation ability, and does not interfere with the normal cornea [2]. Posterior chamber PIOLs are inserted into the eye through a corneal incision and placed in the ciliary sulcus. Globally, the most common forms of this type of intraocular lens are the implantable collamer lenses (ICL; STAAR Surgical, Monrovia, CA). The most commonly used ICL model is the Visian V4c (STAAR Surgical) since it prevents aqueous fluid flow blockade through its central hole design and therefore does not require a peripheral iridotomy as other ICL models do [3]. Although the safety and efficacy of V4c implantation has been recently demonstrated, all previous studies employed classic operation methods; following the corneal incision, ophthalmic viscosurgical devices (OVDs) were injected into the anterior chamber, and then, the intraocular lens was implanted [4–9]. This approach is based on the protective effect to the corneal endothelial cells and other tissues in the eye [10, 11]. However, in our long-term clinical experience, the main disadvantage of this method is the high intraocular pressure (IOP) in the early postoperative period. Although the temporary IOP increase following surgery is tolerable for most eyes, an excessive IOP after surgery greatly affects patient comfort and could even lead to severe corneal edema, pain, and ischemic anterior optic neuropathy [12]. The early IOP rise following V4c implantation is mainly related to the incomplete removal of the viscoelastic agent. However, the influence of different surgical methods on postoperative viscoelastic agent residue and early IOP rise is unknown. Therefore, it is necessary to explore a surgical method that has less influence on IOP. This study aimed to investigate the effects of a modified V4c implantation surgery on the operation time, visual outcomes, and key intraocular safety factors (early IOP and corneal endothelial cell count).

## 2. Materials and Methods

### 2.1. Patients

This was a randomized, prospective, contralateral eye comparison study. The study was approved by the Ethics Committee of the Army Medical Center and was registered in the Chinese Clinical Trial Registry (registration number: ChiCTR1900021704). The study conformed to the principles of the Declaration of Helsinki, and all patients signed informed consent after being informed of all the risks and benefits of the surgery. The inclusion criteria were as follows: 20–40 years old, spherical equivalent ≥ −6.0 D and stable, anterior chamber depth ≥2.8 mm, corneal endothelial cell count ≥2000 cells/mm^2^, normal IOP, and no history of eye surgery. The exclusion criteria were as follows: spherical equivalent <−6.0 D, anterior chamber depth <2.8 mm, corneal endothelial cell count <2000 cells/mm^2^, and ocular diseases such as corneal abnormalities, glaucoma, uveitis, or macular lesions.

### 2.2. Groups and Interventions

In total, 192 eyes from 96 patients were included in this study, and the two eyes from the same patient were randomly assigned to two groups (group 1 and group 2, with 96 eyes in each group). After making the corneal incision, OVDs were first injected into the anterior chamber followed by V4c ICL implantation in group 1, whereas the ICL was first implanted into the anterior chamber followed by OVDs injection in group 2.

### 2.3. Measures of the Outcome

A preoperative examination was performed for all patients at the Army Medical Center (Daping Hospital, Chongqing, China). Visual acuity was measured using the international standard visual acuity chart, which was converted to logarithm of the minimal angle of resolution (logMAR). A noncontact tonometer (Canon, Tokyo, Japan) was used to measure the IOP. Corneal endothelial cells were counted using a corneal endothelium microscope (SP-2000P, TOPCON, Tokyo, Japan). The anterior chamber angle was measured using visante optical coherence tomography (OCT) (Carl Zeiss Meditec, Jena, Germany; 0° and 180° angles were measured. The apex was the scleral spur, and the line length was 500 *μ*m), while the power of the V4c lens was measured using software provided by STAAR Surgical (Monrovia, CA). The size of V4c was determined by the white-to-white distance and the anterior chamber depth. The same instrument was used to measure the corresponding indices following the operation.

### 2.4. Surgical Procedure

All surgeries were performed by the same experienced surgeon (JY), and no adverse events (such as infectious endophthalmitis, corneal endothelium injury, or toric V4c rotation) occurred during or after surgery.

  Procedure for group 1: the pupils were fully dilated before surgery. After injection of sodium hyaluronate (15 mg/mL) into the anterior chamber, a V4c ICL (STAAR Surgical) was implanted via a 3.0 mm corneal incision using an injector cartridge and then placed in the posterior chamber. Next, the ICL was carefully positioned in the ciliary sulcus (toric ICLs were rotated to the marked position based on the degree of rotation calculated before surgery). OVDs were completely washed away using balanced salt solution, and 0.1% cefuroxime (0.1 mL) was injected into the anterior chamber.
  Procedure for group 2: the pupils were fully dilated before surgery. ICL V4c was implanted via a 3.0 mm corneal incision using an injector cartridge, and sodium hyaluronate (15 mg/mL) was injected into the anterior chamber. After that, V4c was carefully placed in the posterior chamber and positioned in the ciliary sulcus (in the case of toric ones, V4c was rotated to the marked position based on the degree of rotation calculated before surgery). The OVDs were completely washed away using the balanced salt solution, and 0.1% cefuroxime (0.1 mL) was injected into the anterior chamber.

Postoperative medications included nonsteroidal anti-inflammatory eye drops, antibiotic eye drops, and artificial eye drops.

### 2.5. Statistical Analysis

All results were expressed as mean ± standard deviation, except for the ratio of the increased IOP and the corneal endothelium loss rate (expressed as a percentage). The IOP and the corneal endothelial cell count at different time points were analyzed using two-way repeated-measures analysis of variance; when there were differences between timepoints, the least significant difference post hoc test was used to compare the differences between timepoints. Pearson's chi-squared test was used to compare the ratio of the increased IOP at 2 hours postoperatively and the corneal endothelium loss rate at 3 months postoperatively. A paired *t*-test was used to compare the operation time, visual acuity, spherical equivalent, and anterior chamber angle (the Wilcoxon rank-sum test was used when the data were not normally distributed). Statistical analysis was performed using SPSS (version 22.0; IBM, Armonk, NY), and *P* < 0.05 was considered statistically significant.

## 3. Results

### 3.1. Operation Times

The operative time of group 1 was 4.68 ± 0.77 min and that of group 2 was 3.07 ± 0.61 min. There was a significant difference between the two groups (*P* = 0.002 < 0.05).

### 3.2. Visual Outcomes

All patients were followed up for 3 months postoperatively, and no patient was lost to follow-up. The visual outcomes before surgery and 3 months after surgery in both the groups are given in Table 1.

### 3.3. Safety and Efficacy

**Figure 1:**
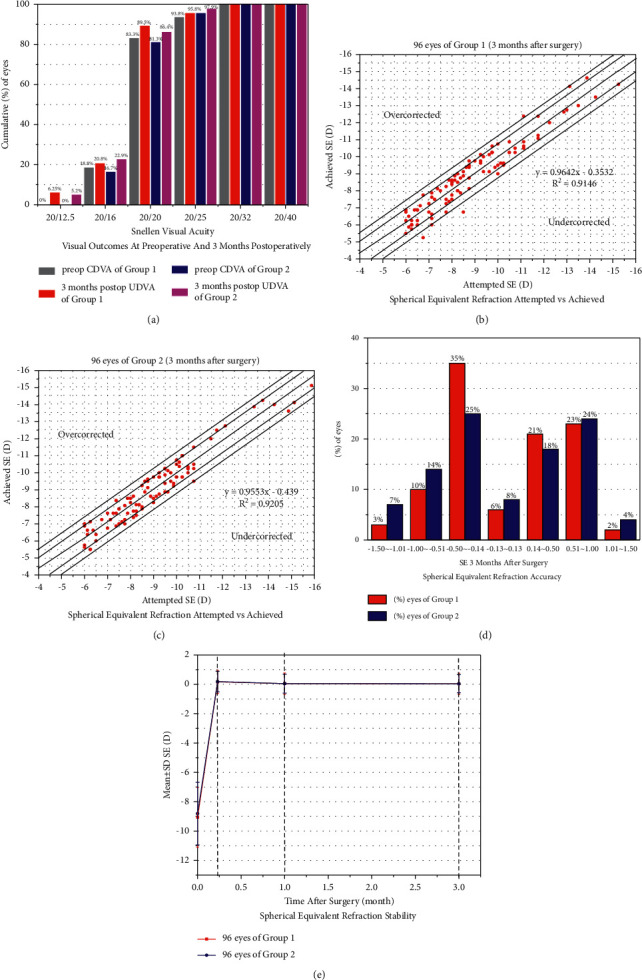
(a) Visual outcomes at 3 months postoperatively. (b) Spherical equivalent refraction attempted vs. achieved in group 1. (c) Spherical equivalent refraction attempted vs. achieved in group 2. (d) Spherical equivalent refraction accuracy. (e) Spherical equivalent refraction stability. UDVA, uncorrected distance visual acuity; CDVA, corrected distance visual acuity; SE, spherical equivalent; D, diopters; SD, standard deviation.

At 3 months after surgery, the safety index (postoperative corrected distance visual acuity (CDVA)/preoperative CDVA) was 1.21 ± 0.23 in group 1 and 1.17 ± 0.19 in group 2, and the efficacy index (postoperative uncorrected distance visual acuity (UDVA)/preoperative CDVA) was 1.33 ± 0.18 in group 1 and 1.20 ± 0.14 in group 2. Among all eyes, 89.5% had a postoperative UDVA of 20/20 or better in group 1 and 86.4% had a postoperative UDVA of 20/20 or better in group 2. Furthermore, 100% had a postoperative UDVA of 20/40 or better in group 1 and 100% had a postoperative UDVA of 20/40 or better in group 2 (Figure 1(a)).

### 3.4. Predictability and Stability

The attempted versus achieved spherical equivalent (SE) corrections are shown in a scatter plot (Figures 1(b) and 1(c)). At the last follow-up, 64% and 51% of the eyes were within ±0.50 D and ±0.50 D in groups 1 and 2, respectively; 5% and 11% of the eyes were beyond ±1.0 D of the attempted refraction of groups 1 and 2, respectively (Figure 1(d)). The refraction remained stable during the follow-up period (Figure 1(e)).

### 3.5. Corneal Endothelial Cell Count

**Figure 2:**
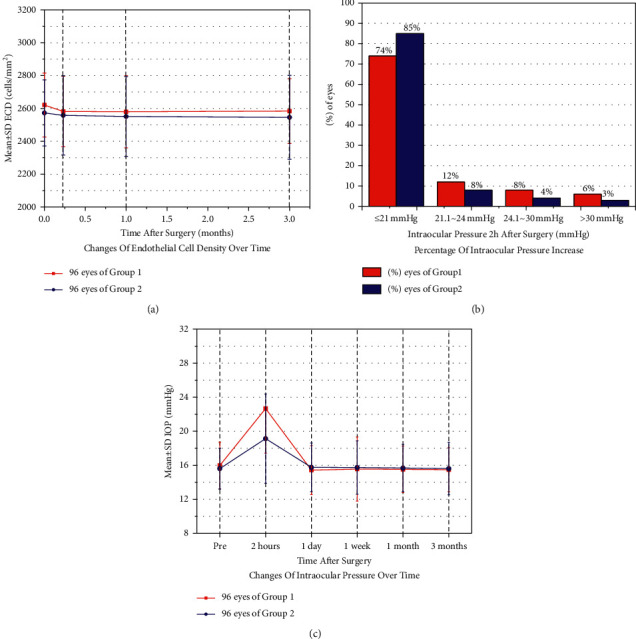
(a) Changes in corneal endothelial cell count over time. (b) Percentage of intraocular pressure increase at 2 hours following surgery. (c) Changes in intraocular pressure over time. SD, standard deviation; ECD, endothelial cell density; IOP, intraocular pressure.

There were no significant differences between the two groups before surgery, 1 week following surgery, 1 month following surgery, or 3 months following surgery (all *P* > 0.05). The corneal endothelium loss rate at 3 months was 0.3% and 1.0% in groups 1 and 2, respectively, with no significant difference (*P* = 0.736 > 0.05). A comparison of the corneal endothelial cell counts is given in Table 2. Changes in the corneal endothelial cell count over time are shown in Figure 2(a).

### 3.6. Intraocular Pressure

There were significant differences between IOP measured 2 hours following surgery of the two groups (*P* = 0.026 < 0.05), but there were no significant differences between the two groups before surgery, 1 day following surgery, 1 week following surgery, 1 month following surgery, or 3 months following surgery (all *P* > 0.05). The rate of increase in IOP (≥21 mmHg) was significantly different between the two groups at 2 hours following surgery (26.0% in group 1 and 15.0% in group 2; *P* = 0.019 < 0.05). A comparison of the IOP between groups is given in Table 3. The percentage of IOP increase at 2 hours following surgery is shown in Figure 2(b), and changes in IOP over time are shown in Figure 2(c).

### 3.7. Anterior Chamber Angle

The anterior chamber angle of group 1 was 54.74 ± 5.59° preoperatively and 34.63 ± 4.81° 2 hours postsurgery and 54.68 ± 5.66° preoperatively and 34.57 ± 4.56° 2 hours postsurgery in group 2. There were significant differences in the anterior chamber angle 2 hours after surgery compared with that before surgery in both groups (*P* = 0.014 < 0.05 and *P* = 0.029 < 0.05, respectively). There were no significant differences between groups at the same time point (*P* = 0.793 > 0.05 and *P* = 0.674 > 0.05, respectively).

## 4. Discussion

The clinical results of UDVA and CDVA 3 months following surgery in both groups indicated that both ICL implantation methods were safe and effective. Furthermore, the clinical results of SE at 3 months postoperatively in both groups confirmed that both ICL implantation methods had good predictability. Moreover, the stability of the two ICL implantation methods was confirmed by the change in SE over time.

Considering the literature on ICL implantation, little attention has been paid to the operative time. One study by Ganesh et al. compared the influence on operation time of the two different OVDs used in V4c implantation: hyaluronic acid (1%) and hydroxypropyl methylcellulose (HPMC) (2%), and they demonstrated that the total operation time of the 1% sodium hyaluronate group was shorter than that of the 2% HPMC group. This was possibly due to the relatively higher viscosity of 1% sodium hyaluronate that facilitated its complete removal during aspiration, thus significantly reducing the operative time [13]. Our study found that the operation time of group 2 was 1.61 min shorter than that of group 1. Compared with first injection of OVDs, the subsequent steps of first implantation of a V4c ICL only necessitate the removal of the OVDs in front of the IOL instead of behind it. In other words, the removal of the OVDs became easier and faster, and the total operation time was significantly reduced. The rapid removal of OVDs during the operation not only significantly reduces the operation time but also reduces the interference to the intraocular tissues, especially the natural lens, and effectively protects the tissue in the eye and reduces the risk of cataract.

V4c implant surgery is an intraocular surgery, and the effect of implantation on corneal endothelial cells is substantial. Alfonso et al. found that the corneal endothelial cell count decreased by 8.5% (*P* = 0.000 < 0.001) 6 months after surgery, and the postoperative average corneal endothelial cell count was 2533/mm^2^, which is still much higher than the critical functional density [14]. Another study by Cao et al. observed that the corneal endothelial cell count decreased by 2.0% 6 months after surgery compared with that before surgery (*P* = 0.000 < 0.001), and no complications related to corneal endothelial injury were detected during follow-up [15]. Furthermore, Kamiya et al. implanted an intraocular lens with an anterior chamber depth of less than 3 mm and found that the postoperative corneal endothelial cell loss rate was 0.2 ± 8.7%, confirming the safety of V4c implantation even in the case of a shallow anterior chamber. They also summarized the statistical results from other studies that examined corneal endothelial cell count after ICL implantation and found that the postoperative corneal endothelial cell loss rate was between −1.1% and 8.5% [16]. In our clinical experiment, the corneal endothelial cell loss rate at 3 months in the two groups was 0.3% and 1.0%, respectively. These findings suggest that the two surgical methods have similar effects on corneal endothelial cells, and the surgical method of implanting the V4c first did not cause significant damage to the corneal endothelial cells. Alternatively, compared with the reasonable use of OVDs, extensive experience and a delicate operation were more significant for the protection of the endothelial cells.

IOP, as one of the key intraocular safety factors, must also be the focus of intraocular surgery. At present, an elevated IOP after ICL implantation is very common. Gonzalez-Lopez et al. studied the IOP in 100 eyes at 1 day following V4c implantation and found that the IOP of 5 eyes was ≥22 mmHg. They believed that residual OVDs blocking the trabecular mesh and the central hole of the V4c were responsible for this increase [17]. Another study by Senthil et al. assessing 638 eyes from 359 people found that the IOP increased in 5 cases (≥22 mmHg) 1 day following V4c implantation due to residual or excessive filling of the anterior chamber (accounting for 15% of all causes of IOP increase) and found that short-term use of antiglaucoma drugs could reduce the IOP [18]. Almalki et al. studied the causes of increased IOP following V4c implantation. They found that at 4–6 h after surgery, closure of the anterior chamber angle was caused by a high vault in 10.3% of cases. After 1 day following the operation, there were residual OVDs in 39.7% of cases, whereas local hormone drug use was responsible for increased IOP in 37.9% of cases at 2–4 weeks following surgery [19]. In our study, we found that the increase in IOP in both groups occurred at 2 h following surgery, and the rate of increase in group 2 (≥21 mmHg) was lower than that in group 1. In most patients (60%), IOP can be restored to the normal range following the application of miotics. In fact, the IOP was normal 1 day following the operation and remained stable for 1 week, 1 month, and 3 months following the operation. The anterior chamber angle in both groups was significantly reduced 2 hours following the operation compared to that before the operation. The results of the IOP and the anterior chamber angle showed that the narrowing of the anterior chamber angle following V4c implantation was an important factor leading to an early postoperative IOP increase. Residual OVDs still existed in the surgical method of implanting the V4c ICL first, which obstructed the trabecular mesh (the outflow channel of aqueous humor circulation) and led to an increase in IOP. However, compared with the surgical method of injecting OVDs first, the removal of OVDs was more rapid and thorough in group 2, and the operation time was shorter, thus reducing the interference to the aqueous humor circulation in the eye; therefore, the rate of IOP increase in the early postoperative period was lower.

There were certain limitations to this study. First, the follow-up time was not long enough, and further follow-up and collection of relevant clinical indicators are needed to verify the long-term clinical effect of the surgical method with implantation of the V4c lens. Second, the operation method of V4c implantation required the operation to be performed delicately; therefore, the operator requires extensive surgical experience and exquisite operation skills, which are not suitable for beginners or inexperienced operators.

## 5. Conclusions

In summary, the two ICL implantation methods had similar clinical outcomes and effects on the corneal endothelial cell count. Additionally, implantation of an intraocular lens prior to injecting OVDs reduces the operation time and lowers the rate of IOP rise in the early postoperative period, making it a safe and effective surgical method for ICL implantation.

## Figures and Tables

**Table 1:** Visual outcomes before and 3 months after surgery in both groups.

Group	UDVA (logMAR)		CDVA (logMAR)		SE (D)	
	Pre	3 months	Pre	3 months	Pre	3 months
Group 1	1.49 ± 0.24 ^*∗*^	−0.04 ± 0.08^ǂ^	−0.03 ± 0.09 ^*∗*^	−0.05 ± 0.08^ǂ^	−8.81 ± 2.13 ^*∗*^	0.25 ± 0.62^ǂ^
Group 2	1.53 ± 0.23	−0.06 ± 0.09	−0.05 ± 0.10	−0.08 ± 0.07	−9.06 ± 2.04	0.32 ± 0.72
*Z*	−1.483	−0.049	−0.090	−0.548	−0.995	−0.111
*P*	0.138	0.961	0.928	0.584	0.320	0.912

^*∗*^Group 1 vs. group 2 at preoperative, *P* > 0.05. ^ǂ^Group 1 vs. group 2 at 3 months after surgery, *P* > 0.05. UDVA, uncorrected distance visual acuity; CDVA, corrected distance visual acuity; logMAR, logarithm of the minimal angle of resolution; SE, spherical equivalent; D, diopter.

**Table 2:** The comparison between the corneal endothelial cell count (cells/mm^2^) of the two groups.

Time	Group		95% CI for difference		*P*
	Group 1	Group 2	Lower limit	Upper limit	
Preoperative	2591.25 ± 230.01 ^*∗*^	2572.40 ± 202.46	−76.164	38.476	0.516
1 week	2581.70 ± 215.65 ^*∗*^	2557.66 ± 242.81	−80.914	32.831	0.403
1 month	2579.50 ± 220.45 ^*∗*^	2550.00 ± 243.94	−85.574	28.429	0.322
3 months	2583.98 ± 198.15 ^*∗*^	2545.50 ± 256.39	−96.758	19.799	0.193

^*∗*^Group 1 vs. group 2 at each time point, *P* > 0.05. CI, confidence of interval.

**Table 3:** Comparison of the intraocular pressure (IOP) (mmHg) between the two groups.

Time	Group		95% CI for difference		*P*
	Group 1	Group 2	Lower limit	Upper limit	
Preoperative	15.97 ± 2.74^ǂ^	15.60 ± 2.40	−1.138	0.445	0.387
2 hours	22.67 ± 5.24 ^*∗*^	19.13 ± 5.25	−2.743	0.325	0.026
1 day	15.43 ± 2.88 ^ǂ^	15.76 ± 2.86	−1.174	0.541	0.465
1 week	15.55 ± 3.76 ^ǂ^	15.74 ± 3.13	−0.626	1.053	0.615
1 month	15.52 ± 2.74 ^ǂ^	15.68 ± 2.78	−0.651	0.999	0.676
3 months	15.48 ± 2.59 ^ǂ^	15.61 ± 3.09	−0.557	0.813	0.711

^*∗*^Group 1 vs. group 2 at 2 hours after surgery, *P* < 0.05. ^ǂ^Group 1 vs. group 2 at other time points, *P* > 0.05. CI, confidence of interval.

## Data Availability

The data used to support the findings of this study are available from the corresponding author upon request.
